# Enhancing identification of nonaffective psychosis in register-based studies

**DOI:** 10.1038/s41537-024-00444-6

**Published:** 2024-02-19

**Authors:** Minna Holm, Kimmo Suokas, Emmi Liukko, Maija Lindgren, Petri Näätänen, Jukka Kärkkäinen, Raimo K. R. Salokangas, Jaana Suvisaari

**Affiliations:** 1 Finnish Institute for Health and Welfare, Equality Unit, Helsinki, Finland; 2 Finnish Psychiatric Association, Helsinki, Finland; 3 Tampere University, Faculty of Social Sciences, Tampere, Finland; 4 Finnish Institute for Health and Welfare, Data and Analytics Unit, Helsinki, Finland; 5 Department of Psychiatry, University of Helsinki and Helsinki University Hospital, Helsinki, Finland; 6 County of Satakunta, Psychiatry of Wellbeing services, Satakunta, Finland; 7 Department of Psychiatry, University of Turku, Turku, Finland

**Keywords:** Psychosis, Schizophrenia

## Abstract

The Finnish Quality of Psychosis Care Register assesses nonaffective psychosis (NAP) care, acknowledging treatment outside specialized psychiatric services. This approach, while providing a holistic view, raises concerns about diagnostic inaccuracies. Here, we studied situations where the register-based diagnosis might be inaccurate, and whether the first episode can be reliably identified using a 14-year wash-out period. People with first register-based NAP (ICD-10 F20-F29) between years 2010 and 2018 and without NAP diagnoses in 1996–2009 were identified from the Care Register for Health Care. A diagnosis of NAP was deemed unreliable before age 7, when dementia preceded NAP diagnosis, and when a NAP diagnosis had been assigned at admission or during psychiatric hospitalization but was not confirmed by discharge diagnosis. Despite a 14-year follow-back the first register diagnosis may miss the first treatment episode in older patients. Register-based studies on psychotic disorders should pay attention to exclusion criteria and to the definition of treatment onset.

## Background

Nationwide population-based healthcare registers are invaluable resources for research and a data source for national healthcare performance indicators. Currently, we utilize them to develop a quality-of-care register for psychotic disorders in Finland. Comprehensive registers especially in the Nordic countries have unique strengths, including full representativeness with no loss to follow-up except for emigration. However, register-based research is not without limitations. Diagnoses are not set for research purposes and hence diagnostic accuracy in the registers is varying^1,2^.

The Finnish Quality of Psychosis Care Register (FQPCR) aims to monitor and evaluate the quality of care and clinical and social outcomes in individuals with a history of treatment for schizophrenia or other primary psychotic disorders (referred in this paper as nonaffective psychoses [NAP]). With these aims, the accuracy of cohort identification algorithm is crucial. Since the psychosocial treatment in the early years should be particularly intensive^3^, being able to identify first psychotic episode is also important. For this purpose, register-based research commonly uses a wash-out period of several years, but its length varies.

The Finnish Quality of Psychosis Care register includes information on both primary care and specialized care from all medical specialties. This is important for monitoring comprehensively the quality of clinical care pathways (e.g. the transfer of care to primary care) and the quality of treatment of physical health problems. However, this also means that the first diagnosis of NAP may have occurred outside psychiatric services, for example in primary care, and these diagnoses may be less reliable than those assigned in psychiatric services.

We were especially interested in three situations where NAP diagnosis may be unreliable: children under the age of seven, people with dementia diagnosis before the first NAP diagnosis and people who have received NAP diagnosis as a preliminary diagnosis at admission to inpatient treatment or during hospital treatment. Nonaffective psychotic disorders are very rare in children under 13 years^4,5^. Diagnosing psychosis in children is complex because conceptualizing psychotic experiences is difficult for young children, and differentiation from other disorders, such as autism or mood disorders, is challenging^6–8^. A Danish validation study showed that the diagnosis of very early onset schizophrenia had low accuracy especially if the diagnoses were made in outpatient care^9^. Prior research has not granted an age cut-off from which the diagnoses can be considered to be more reliable, but the age of around six or seven was estimated based on the prior literature^6,9^. Psychotic symptoms are common in people with dementia (18), but in this group, psychotic symptoms typically result from dementia rather than from new NAP. Their exclusion is important because good quality of care and organization of services are different for people with dementia with psychotic symptoms and for people with NAP. At discharge, the diagnosis is based on all the information acquired during the hospital treatment, whereas diagnoses given earlier during the hospitalization are based on the information acquired by that time. During hospital treatment, a within or between hospital transfer causes a new register entry in Finland, and a diagnosis that can be preliminary is marked. Another example of preliminary diagnoses are diagnoses given in the emergency department^10,11^.

The aim of the present study is to improve the algorithm to identify NAP from health care registers by detecting factors that should be considered. To address the aim we used a cohort of all people with first register-based NAP between the years 2010 and 2018 and without NAP diagnoses in 1996-2009 in the Care Register for Health Care (CRHC). We also examined how well the age of first register-diagnosis represents the age of onset by comparing age and diagnostic groups with the proportions of prior antipsychotic purchases and prior specialized reimbursement rights for medications due to severe psychosis or other severe mental disorder. In addition, we compared the recurrence of NAP diagnosis and antipsychotic medication use in patient groups identified from different treatment facilities and the effect of exclusions to the registry patient population characteristics.

## Results

### The composition of the cohort

The most common first psychosis diagnostic entry in the CRHC was unspecified psychosis not due to a substance or known physiological condition (48%) and the least common was schizotypal disorder (3%, Additional file 2, Supplementary Table 1). The first diagnosis was received in primary health care by 10 106 (21%), in psychiatric outpatient care by 10 973 (22%), in other specialized health care by 1 321 (3%) and at hospital discharge, during hospitalization or one day before by 26 764 (54%).

### Psychiatric diagnoses before and age distribution at the first NAP diagnosis

**Fig. 1:**
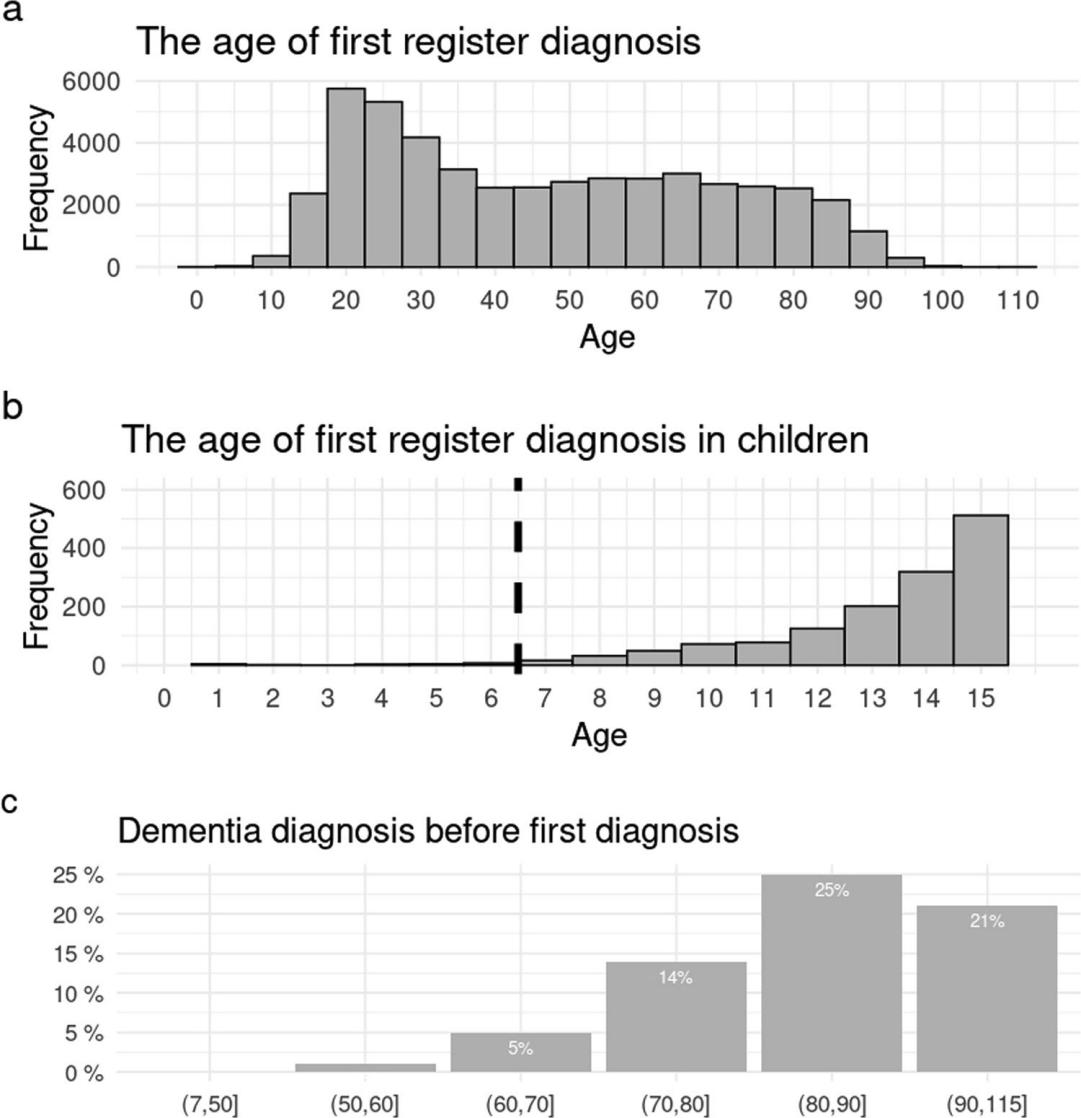
Age distribution of first Care Register for Health Care diagnosis of nonaffective psychosis and the probability of dementia diagnosis before the first nonaffective psychosis diagnosis. Age distribution of the first diagnosis of nonaffective psychosis is shown **a** in the whole cohort and **b** in children. The dashed line represents the proposed cut-off age. **c** represents the probability of dementia diagnosis before the first nonaffective psychosis diagnosis by age.

The mean age at the first NAP diagnosis was 47 years but the distribution was right-skewed. Altogether, 2757 (6%) had received their first diagnosis before the age of 18 and 391 (0.8%) before the age of 13 (see Fig. 1a and b). NAP diagnosis had been given to 19 persons before the age of 7. Only few of them (*n* < 5) had received the diagnosis as the discharge diagnosis from a psychiatric hospitalization and five (26%) received another NAP diagnosis during two-year follow-up. The first register-diagnosis had been received by 16 222 (33%) at or after age 60.

Before the first NAP diagnosis, 31 361 (64%) had received any psychiatric (F00-F99) diagnosis in health care. The most common diagnoses were nonpsychotic depression (*n* = 15 941, 32%) and anxiety disorder (*n* = 14 656, 30%, Additional file 2, Supplementary Table 2). Bipolar disorder diagnosis had been received by 3 416 (7%), depressive disorder with psychotic symptoms by 3 459 (7%) and substance-use induced psychosis by 2587 (5%). Before their first psychosis diagnosis, 2 278 (5%) had received dementia diagnosis. The probability of dementia diagnoses rose sharply with age and at age of 80–90 one in four persons had received dementia diagnosis before the first NAP diagnosis (Fig. 1c).

Based on the inspection of age and previous diagnoses, we decided to use the first diagnosis before the age of 7 and dementia diagnosis before the first register diagnosis as exclusion criteria in the following analyses.

### Stability of preliminary NAP diagnosis within first hospitalization

**Fig. 2:**
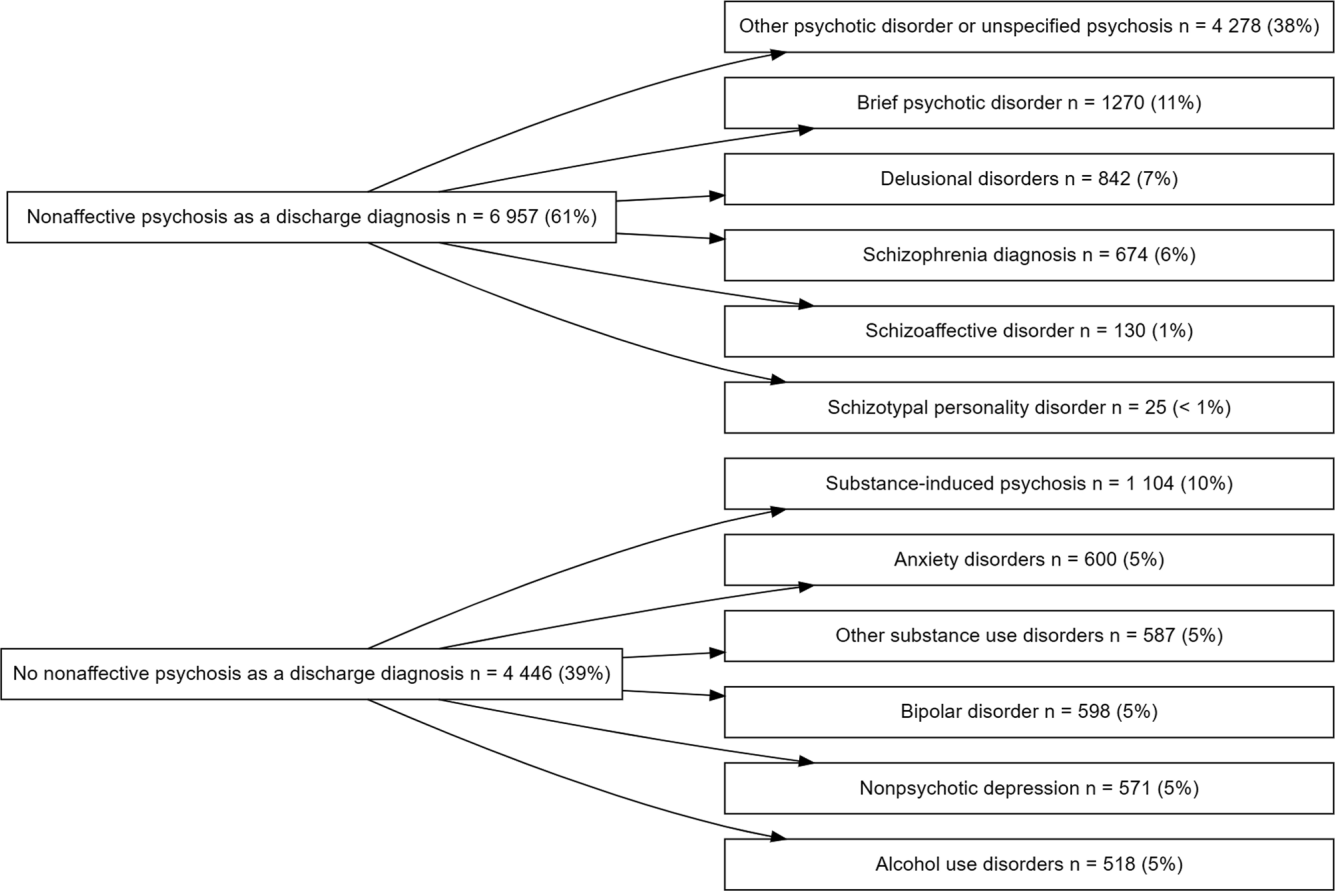
The discharge diagnoses in people who have received their first nonaffective psychosis diagnosis in emergency setting at the beginning of psychiatric hospitalization.

Of the people who received their first NAP diagnosis at emergency setting one day before or on the day of psychiatric hospital admission (*n* = 11 708), 61% received NAP diagnosis as a discharge diagnosis, the most common diagnoses being other or unspecified psychotic disorder or brief psychotic disorder (see Fig. 2). If the person did not receive NAP diagnosis, the most common discharge diagnoses were substance use disorders, especially substance-induced psychotic disorder, as well as bipolar disorder, nonpsychotic depression and anxiety disorders.

Of people who received their first psychosis diagnosis during hospital treatment or one day before admission excluding the people who had received the diagnosis at emergency setting (*n* = 4 821), 63% received a NAP diagnosis as their discharge diagnosis (Additional file 2, Supplementary Table 3A). If the person did not receive a NAP diagnosis as discharge diagnosis, the most common diagnoses were psychotic and nonpsychotic depression as well as alcohol use disorders and substance induced psychoses (Additional file 2, Supplementary Table 3B).

### Effect of the health care setting where the diagnosis had been assigned

Next, we investigated the effect of the health care setting where the diagnosis had been assigned on the probability of receiving a NAP diagnosis the second time and purchasing antipsychotics within two-year follow-up. The people whose first NAP diagnosis was received during psychiatric hospitalization were divided to people who received and did not receive a NAP diagnosis as a discharge diagnosis.

**Fig. 3:**
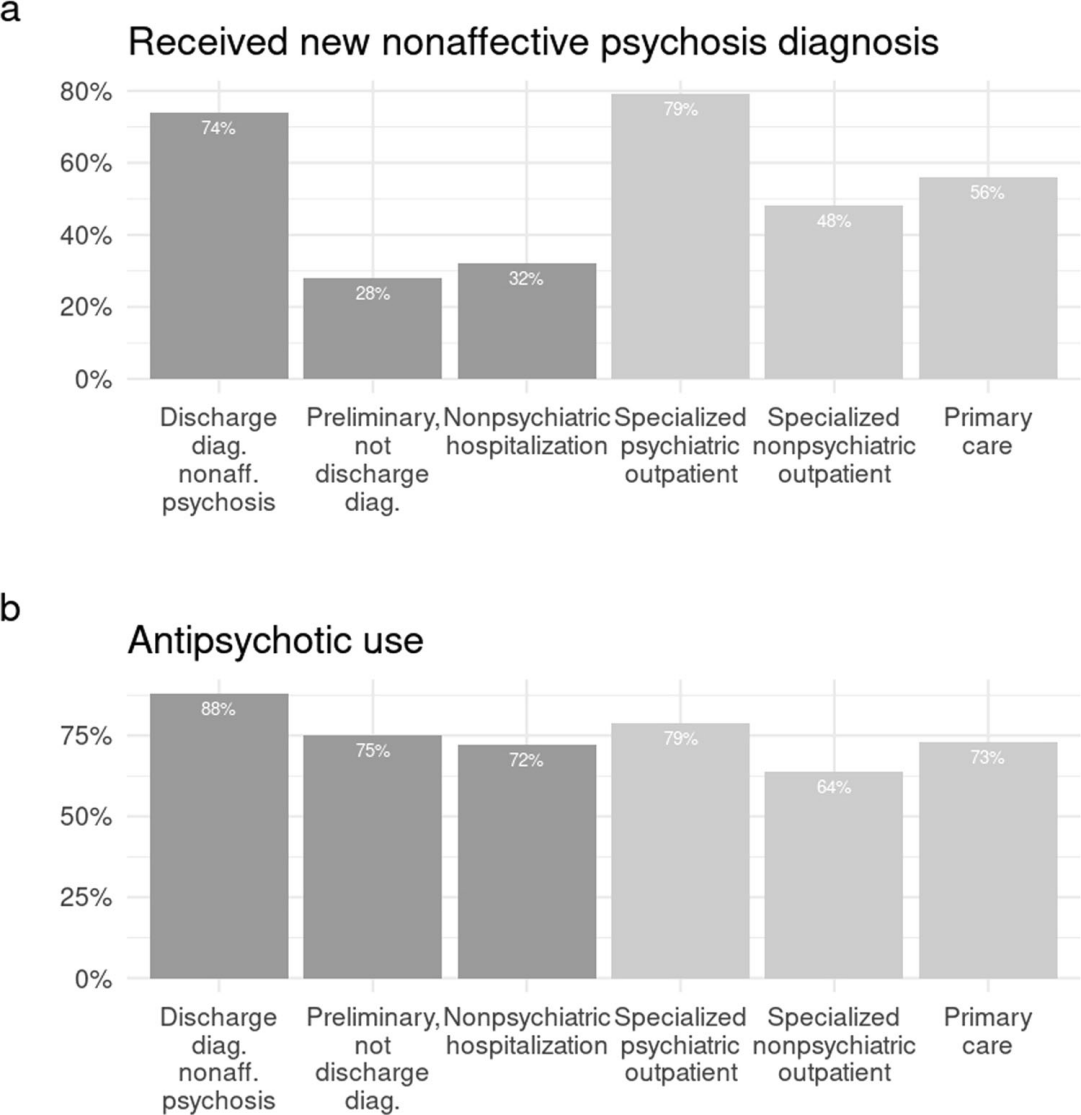
The outcomes at 2-year follow-up. The probability **a** to receive another nonaffective psychosis diagnosis and **b** to purchase antipsychotics at 2-year follow-up.

The people who had received their first NAP diagnosis in psychiatric outpatient care and people who had received NAP diagnosis as a discharge diagnosis were the most likely to receive another NAP diagnosis (see Fig. 3). The people who had received their first NAP diagnosis during nonpsychiatric hospitalization or who had not received NAP diagnosis as a discharge diagnosis at the end of psychiatric hospitalization were the least likely to receive another NAP diagnosis. Antipsychotic purchases were common in all groups identified from different treatment settings but the people who had received NAP diagnosis as a discharge diagnosis from psychiatric hospitalization had the highest percentage of purchasing antipsychotics during the two-year follow-up.

When we looked at the frequency of other psychotic disorder diagnoses in people who had not received another NAP diagnosis, people who had received their first NAP diagnosis within psychiatric hospitalization but had not received NAP diagnosis as a discharge diagnosis were most often diagnosed as having substance-induced psychotic disorder, bipolar disorder or psychotic depression (Additional file 2, Supplementary Fig. 1). In others, these diagnoses were markedly less common.

The differences between diagnostic groups, based on the first diagnosis, in receiving another NAP diagnosis were not as large as between treatment facilities (Additional file 2, Supplementary Fig. 2). People with schizotypal disorder or International Classification of Primary Care [ICPC]-2NAP diagnosis had purchased antipsychotics less frequently than other diagnostic groups.

Finally, we performed logistic regression models to examine how different factors together predict new NAP diagnoses and purchasing antipsychotics during the follow-up (Additional file 2, Supplementary Table 4). Old age decreased the probability of receiving another NAP diagnosis. However, it should be acknowledged that mortality in two-year follow-up is relatively high especially in people over 80 years old (23% in the age group of 80-90 and 41% in people over 90 years old). In addition, the first NAP diagnosis received during psychiatric hospital care without confirmation by the discharge diagnosis (OR = 0.11, 95% CI = 0.10–0.11, reference: the first diagnosis from psychiatric outpatient care) as well as the first diagnosis from nonpsychiatric hospital care (OR = 0.20, 95% CI = 0.18–0.21) were the least likely to be followed by another NAP diagnosis. In addition, people who received the first diagnosis in primary care had lower probability of receiving another diagnosis (OR = 0.41, 95% CI = 0.38–0.44).

The group who received their first diagnosis as a discharge diagnosis from psychiatric hospital care (OR = 2.05, 95% CI = 1.91–2.20) were the most likely to purchase antipsychotics, whereas the people whose first diagnosis was schizotypal disorder (OR = 0.51, 95% CI = 0.45–0.58) or based on ICPC-2 (OR = 0.58, 95% CI = 0.52–0.64) were the least likely.

Because of low stability of preliminary diagnoses during hospitalization as well as decreased probability of receiving new NAP diagnoses and increased probability of receiving other psychosis diagnoses during follow-up if preliminary NAP diagnosis had not been confirmed by discharge diagnosis, we decided not to use the NAP diagnoses received one day before or during hospitalization, and to use discharge diagnoses instead.

### The effects of employing the exclusion criteria

**Fig. 4:**
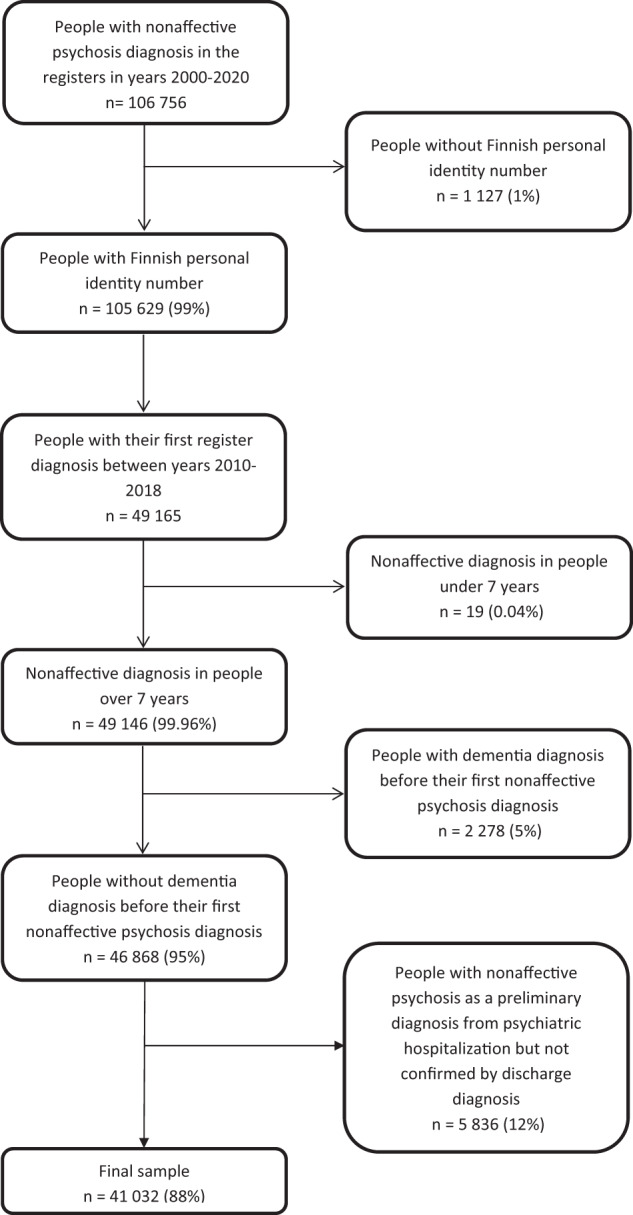
Flow-chart of how the final cohort was formed.

Based on the presented results, we decided to employ the following exclusion criteria in the quality register for NAP disorders (Fig. 4):

- People with their diagnosis under age 7
- People with dementia diagnosis before their first NAP diagnosis
- People whose diagnosis had only been assigned during psychiatric hospitalization, but the diagnosis was not confirmed by discharge diagnosis.

Only 19 persons were excluded due to age but 12% were excluded because they had received NAP diagnosis during psychiatric hospitalization that had not been confirmed by hospital discharge diagnosis. Originally, the sample size was 49,146 and after the exclusion 41 032. Applying the exclusion criteria lowered the mean age of the cohort from 47 years to 44 years and increased the probability of receiving new NAP diagnosis after the first diagnosis (Table 1). Diagnostic distribution or sex proportions were not affected.

**Table 1:** Comparison of the original and the final sample on diagnostic characteristics.

	Original sample		Final sample^a^	
Characteristic	n or Mean	% or SD	n or Mean	% or SD
n and % of the original	49165	100	41032	83
Women	25211	51	20716	50
Age, Mean and SD	47	23	44	22
Original diagnosis				
Other psychotic disorder or unspecified psychosis	23790	48	20297	49
Delusional disorders	8377	17	6593	16
Brief psychotic disorder	6313	13	5129	12
Schizophrenia	4961	10	4147	10
ICPC-2 nonaffective psychosis diagnosis	4317	9	3515	9
Schizoaffective disorder	1565	3	1412	3
Schizotypal disorder	1340	3	1266	3
Probability of new nonaffective psychosis diagnosis	29751	61	29049	71

^a^after employing the exclusion criteria presented.

### Uncertainties in defining the age at onset

With our final NAP sample, we returned to the question of identifying the age at treatment onset. To evaluate if the first register diagnosis represents the age of onset of the illness or treatment, we examined the proportion of people with a special reimbursement right to antipsychotic medication before the first diagnosis. Of individuals with the first CRHC diagnosis of NAP in 2010-2018, 9 810 (20%) had received the special reimbursement right before the first NAP diagnosis (see Supplementary Table 5 for details).

Secondly, we investigated whether treatment had been started before the first diagnosis of NAP by investigating antipsychotic medication purchases. Antipsychotic medication had been purchased by 15 838 (39%) at least one year before the first register-based diagnosis. Of them, 7 980 (50%) had a special reimbursement right for antipsychotics before the first CRHC diagnosis, whereas 7858 (50%) had been using antipsychotics without a reimbursement right, suggesting symptomatic treatment before the first diagnosis.

**Fig. 5:**
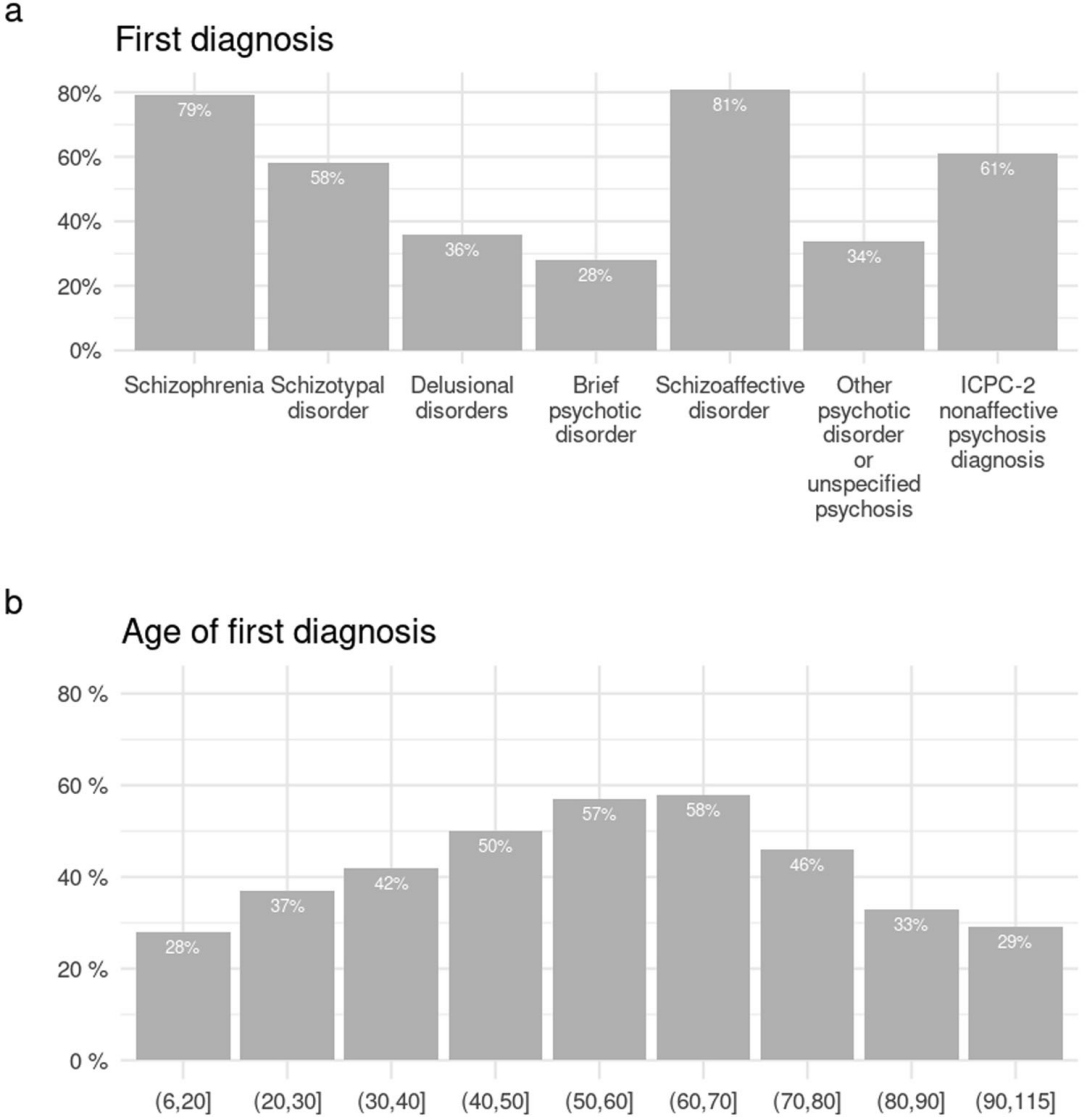
The proportion of people with marks of psychotic episodes or treatment before the first NAP diagnosis in the Care Register for Health Care, indexed by either special reimbursement right prior to the first diagnosis or antipsychotic use at least 1 year before the first diagnosis. The proportions are shown **a** by first diagnosis and **b** by age of first diagnosis.

The probability of either special reimbursement right prior to the first diagnosis or antipsychotic use at least one year before the first diagnosis was highest in people whose first diagnosis was schizophrenia and schizoaffective disorder and at the age group of 50-80 years (Fig. 5). Overall, these results suggest that even with a 14-year follow-back time, a new NAP diagnosis may not always indicate first-episode psychosis. This was especially true for elderly patients.

## Discussion

We identified factors that should be considered when identifying people with NAP in register-based studies and observed a 12% reduction in the size of the sample after excluding these potentially unreliable cases. This is of importance because often register-based studies are based on a simple rule of whether NAP diagnosis can be found in the health-care register. Based on the results of the current study, we ended up excluding from the register the diagnoses assigned to children under age 7, people with dementia diagnosis before their first NAP diagnosis and preliminary diagnoses that had only been assigned during admission or psychiatric hospitalization without confirmation by discharge diagnosis.

The aim of the Quality of Psychosis Care register is to evaluate whether the treatment of NAP is provided according to the current care guidelines. Since the guidelines for treating, for example, affective or substance-induced psychotic disorders are different from NAP, diagnostic accuracy is important. The exclusion of people with only preliminary diagnosis had the most marked effect on the sample size. The exclusion of people with prior dementia diagnosis decreased the mean age of the cohort. The exclusions increased the probability of receiving another psychosis diagnosis during a two-year follow-up, suggesting an improvement in the reliability of the identified diagnoses.

The exclusion of the diagnoses received before the age of seven was based on the previous results that NAP diagnoses in young children have been unreliable^8,9^, and the finding that in the present study, only a small proportion of people who had been diagnosed with NAP under the age of seven had received another NAP diagnosis after the first diagnosis in the registers. In addition, the diagnosis had rarely been assigned as a hospital discharge diagnosis, which could be considered to be more reliable^9^. However, it is possible for these people to be included in the register at or after the age of seven.

When examining previous diagnoses, we noticed that as many as 5% had received dementia diagnosis before the first registered diagnosis, and co-occurrence of dementia was high in people over 70 years. We ended up excluding the people with prior dementia diagnosis, as previously done for example by Stafford et al. ^12^, because the emergence of psychotic symptoms typically results from dementia rather than from new NAP.

When we compared the NAP diagnoses assigned during psychiatric hospitalization before the discharge diagnoses to the discharge diagnoses of the hospital period, defined as the last diagnoses of the hospitalization period, we noticed that only approximately 60% received NAP diagnosis at discharge. Based on previous studies^10,11^, we expected that emergency diagnoses are prone to change, but were surprised to notice that also other diagnoses made during psychiatric hospitalization changed as often. However, these diagnoses could have been assigned for example after a brief stay in a medical unit to ensure that the symptoms are not caused by a general medical condition. The people with only preliminary diagnosis of NAP who did not receive the diagnosis at discharge were also less likely than other groups to receive another NAP diagnosis after the discharge and to receive other diagnoses including psychotic symptoms. Therefore, we ended up recommending that only discharge diagnoses from hospital treatments should be used when selecting people with NAP diagnosis from health-care registers.

For a considerable subgroup, the treatment of psychosis predated the first NAP diagnosis. Even 20% of the whole cohort had received special reimbursement right and 40% had purchased antipsychotic at least one year before the first CRHC diagnosis, although the patients had had no treatment contacts in specialized health care for NAP in years 1996-2009. Of the people who received their first diagnosis at the age of 50-70, over half had either special reimbursement right prior to the first diagnosis or antipsychotic use at least one year before the first diagnosis. Majority of these people may have had a psychotic illness earlier, either an affective psychosis or NAP which had been in remission for a long time or had been treated in primary care, from which register information was not available before 2011. Previous affective or substance-induced psychosis diagnoses were common also in CRHC. NAP that had not been treated in specialized health care for a long-time was the most probable in people whose first diagnosis in the present study was schizophrenia or schizoaffective disorder which was supported by the result that majority of them had antipsychotic use or special reimbursement right prior to the first NAP diagnosis. A small proportion of the identified population may have been treated by private health care, which was not covered by CRHC at the time of this study.

People with NAP in Finland are often treated in the specialized health care. However, in some areas, parts of specialized psychiatric health care administratively belong to primary care. Also, the treatment of NAP in people, who do not need specialized health care mostly because of their long history with the illness, may be transferred to primary care. Therefore, data from primary care is important to get a comprehensive picture of treatment of people with NAP in Finland. Primary care diagnoses may have tapped a comprehensive mix of people with NAP. For some of them, the onset of the psychotic disorder may have been prior to the first register-diagnosis, as inferred by the relatively high proportion of special reimbursement right and antipsychotic use before the first diagnosis in people with ICPC-2 diagnosis. Relatively low frequency of another NAP diagnosis and antipsychotic use within follow-up may index that primary care also faces people with lower engagement to health services or less severe illness course, or the reliability of the diagnoses may be lower than in specialized psychiatric health care.

Validation studies on register-based NAP diagnoses have suggested that there are few false positive cases in the register, but sensitivity may be lower^13–15^. However, the validation studies are already 20 years old and have only used register information from specialized services and not from primary care. New validation studies of the psychosis diagnoses especially in the Register of Primary Health Care Visits would be highly valuable.

The strengths of the study include a nationwide database, and the possibility to comprehensively identify people with NAP diagnosis from specialized as well as primary health care. In addition, there was no attrition to follow-up except due to emigration. The limitations include that only variables in the registers could be used, and the rate of recording diagnoses in primary health care general practitioner visits is only 60%^16^. In addition, we did not have information from private or occupational health care. These healthcare sectors are assumed to be relatively rare in the treatment of psychotic disorder in Finland, and there are no private psychiatric hospitals in Finland.

In conclusion, we identified three factors that should be considered when identifying people with NAP in a register-based study: age under seven, dementia diagnosis and preliminary diagnoses during psychiatric hospitalization not confirmed by discharge diagnosis. We also noticed that the first diagnosis of NAP in registers with a 14-year wash-out period may not tap the first treatment episode in people whose first diagnosis was recorded between ages 50 and 70 and especially if their first diagnosis was schizophrenia or schizoaffective disorder. Therefore, we conclude that when a cohort of people with their first NAP episode is selected, the cohort should be limited to those age groups for whom the data of previous diagnoses from the whole adulthood are available. Other information, such as prior antipsychotic use, can also be used to select people for whom the treatment has started around the time of first diagnosis. Because the groups identified from different health care settings (i.e. specialized psychiatric health care, specialized nonpsychiatric health care and primary care) differed, the most comprehensive picture of people with NAP can be reached by using all health care sectors.

## Methods

### Data sources

We used four registers in the present study: Care Register for Health Care (CRHC), Register of Primary Health Care visits, Register for Reimbursements for Prescription Medicines and Register for Special Reimbursement Right (see Supplementary Methods for the full description of the registers). The CRHC and the Register of Primary Health Care visits contain hospital care (from 1996 in the present project), specialized outpatient care (from 1998) and primary health care (from 2011). In many areas, some of the specialized services have been organized by the municipality (registered in the primary care register) and not only by the hospital districts (registered in the CRHC, see https://sotkanet.fi/sotkanet/fi/taulukko?indicator=s3YutNZNNQAA&region=s06NtM7VMwQA&year=sy5zBAA = &gender=t&abs=f&color=f&buildVersion=3.1.1&buildTimestamp=202309010633&order=A). Therefore, including both primary and secondary care registers is vital for the data coverage^17,18^.

The Register for Reimbursements for Prescription Medicines maintained by the Social Insurance Institution includes all reimbursed medication purchases in Finland. The special reimbursement right that entitles to higher reimbursement of the medicine costs for the treatment of severe mental disorder was collected from the Register for Special Reimbursement Right by the Social Insurance Institution. The right can be granted to people with severe and long-term mental disorder, such as, schizophrenia, delusional disorder, bipolar disorder, psychotic depression or other related disorders with psychotic features. Personal identification number enables individual-level linkage of data in the registers.

The quality registry project received permit to use the registry data from the Finnish Institute for Health and Welfare. Because the data is based on national registers only, separate ethical review or informed consent is not required in Finland.

### Study population

The dataset has been created in the Finnish Quality of Psychosis Care register. All people with NAP diagnostic code (International Classification of Diseases [ICD]-10 codes F20-F29 or International Classification of Primary Care [ICPC]-2 codes P72 or P98) between years 2010 and 2020 were selected from the CRHC and the Register of Primary Health Care visits. ICPC-2 diagnostic classification is used also by physicians in a few primary health care units.

Altogether 106 756 individuals with NAP were identified, of whom 1 127 did not have valid Finnish personal identity number and were excluded, because register-based follow-up cannot be reliably performed, and data from different registers cannot be merged. After this exclusion, the sample size was 105 629.

In the present study, we focused on people whose first diagnosis in the CRCH was between years 2010 and 2018 (*n* = 49 165) and excluded people with a NAP diagnosis in years 1996–2009 in the CRHC. The year 1996 was selected because ICD-10 was implemented at that time in Finland. Thus, we had a 14-year (12 years for specialized psychiatric outpatient care) wash-up period before the year 2010. We followed the identified people for two years, and the follow-up ended at the end of year 2020 at the latest.

### Formation of hospital treatment episodes

We used linkage of inpatient care entries to identify entries that were registered as separate but could still essentially be considered belonging to the same hospitalization^19,20^. This was done by first identifying all inpatient care entries between the years 2010 – 2020 from the CRHC for the study sample. If the date of discharge for previous inpatient care entry and the date of entry for the next inpatient visit were the same, these periods were defined to be part of the same hospitalization period and linked together. If a hospitalization period included such separations due to, for example, transfer to another hospital or to another department within the same hospital, date of entry to the first inpatient care facility was considered as the start of the hospitalization period, and the date of discharge from the last inpatient care facility was considered as the discharge date. Only hospitalizations lasting at least one night after linkage were considered as hospitalizations. Shorter stays were classified as outpatient care. Separate entry and discharge dates were extracted for the period of psychiatric care if they differed from the dates for the whole period. The discharge diagnoses were identified as the last diagnosis given in the psychiatric hospitalization. Psychiatric hospitalization was identified by specialty codes of 70, 70F, 70X, 70Z, 74 or 75.

### Antipsychotic purchases and special reimbursement rights

The purchased medications were retrieved from the Register for Reimbursements for Prescription Medicines. Antipsychotics included ATC group N05A, excluding lithium (N05AN01). People with ongoing special reimbursement rights due to severe mental disorders during the years 1995-2019 were identified from the Register for Reimbursements for Prescription Medicines.

### Statistical analyses

Logistic regression analyses were performed with R version 4.1.3. New diagnoses of NAP and antipsychotic purchase within two-year follow-up were predicted by sex, age, the first register-based diagnosis group (exclusive so a person can belong to only one group), the treatment setting of the first diagnosis, and hospital districts (the largest hospital district, The Hospital District of Helsinki and Uusimaa, HUS, the district was divided to Helsinki and other HUS).

### Supplementary information

Supplement

## Data Availability

Restrictions apply to the availability of the data, which were used under license for the current study, and so are not publicly available. Data can be received from https://findata.fi/en/ with the permission of the Finnish Social and Health Data Permit Authority Findata.
