# Supplementary material for: Enhancing identification of nonaffective psychosis in register-based studies
Source: Schizophrenia (Heidelb). 2024 Feb 19;10(1):20. doi: 10.1038/s41537-024-00444-6 (PMC10876989; doi:10.1038/s41537-024-00444-6)
Supplement: Supplementary file 1 — Supplement [file 41537_2024_444_MOESM1_ESM.pdf]

Supplementary Results: Tables and Figures

Supplementary Table 1. The frequency of first diagnoses in the whole cohort as well as by the treatment facility of the first diagnosis

		Whole cohort		Emergeny setting before psychiatric hospitalization[a]		Preliminary diagnosis within psychiatric hospitalization[a]		Discharge diagnosis from psychiatric hospitalization[a]		Nonpsychiatric hospitalization		Specialized psychiatric outpatient[a]		Specialized nonpsychiatric outpatient		Primary care	
Diagnosis	Diagnosis codes	n	%	n	%	n	%	n	%	n	%	n	%	n	%	n	%
Any nonaffective psychosis	F20-F29	49165	100	11708	100	4821	100	6544	100	3691	100	10973	100	1321	100	10106	100
Other psychotic disorder or unspecified psychosis	F29	23790	48	8551	73	2843	59	3484	53	718	19	6025	55	418	32	1689	17
Delusional disorders	F22 or F24	8377	17	1058	9	601	12	957	15	1304	35	1931	18	405	31	2100	21
Brief psychotic disorder	F23	6313	13	2140	18	743	15	1226	19	434	12	919	8	269	20	501	5
Schizophrenia	F20	4961	10	288	2	350	7	511	8	1101	30	954	9	179	14	1499	15
ICPC-2 nonaffective psychosis diagnosis	ICPC-2: P72 or P98	4317	9	109	1	296	6	0	0	122	3	16	0	2	0	3691	37
Schizoaffective disorder	F25	1565	3	80	1	116	2	305	5	133	4	563	5	45	3	312	3
Schizotypal disorder	F21	1340	3	69	1	34	1	125	2	61	2	635	6	25	2	386	4

[a] Hospitalizations and outpatient visits were considered as psychiatric if the specialty was 70,70F,70X,70Z,74 or 75.

Supplementary Table 2. Frequency of the diagnoses before the first nonaffective psychosis diagnosis

Diagnosis	Diagnosis codes	n	%
Nonpsychotic depression	F32, F33 and F34.1 excluding F32.3 and F33.3	15941	32
Anxiety disorders	F40-F43	14656	30
Nonpsychotic alcohol use disorders	F10 excluding diagnoses with fourth digit 4, 5, or 7	6266	13
Personality disorders	F60-F62, F69	4611	9
Behavioral and emotional disorders with onset in childhood and adolescence	F90-F98	4477	9
Other psychoactive substance use	F11-F19 excluding diagnoses with fourth digit 4, 5, or 7	3770	8
Psychotic depression	F32.3, F33.3	3459	7
Other behavioral syndromes associated with physiological disturbances and physical factors excluding eating disorders	F51, F52, F54-59	3451	7
Bipolar disorder	F30-F31, F34.0	3416	7
Mental disorders due to known physiological conditions excluding dementia	F04-F09	2626	5
Substance-induced psychosis	F10-F19 with fourth digit 4, 5, or 7	2587	5
Dementia	F00-03, G30, G31.0	2278	5

Supplementary Table 3. Diagnoses in people who received their first diagnosis as a preliminary diagnosis during psychiatric hospital treatment

A) The discharge diagnoses in people who received nonaffective psychosis diagnosis as a discharge diagnosis

Diagnosis	Diagnosis codes	n	%
Any nonaffective psychosis	F20-F29	2913	63
Other psychotic disorder or unspecified psychosis	F29	1522	33
Delusional disorders	F22 or F24	444	10
Schizophrenia	F20	433	9
Brief psychotic disorder	F23	420	9
Schizoaffective disorder	F25	122	3
Schizotypal disorder	F21	33	1
ICPC-2 nonaffective psychosis diagnosis	ICPC-2: P72 or P98	0	0

B) The discharge diagnoses in people who did not receive nonaffective psychosis diagnosis as a discharge diagnosis

Diagnosis	Diagnosis codes	n	%
No nonaffective psychosis	No F20-F29	1701	37
Psychotic depression	F32.3, F33.3	268	6
Nonpsychotic depression	F32, F33 and F34.1 excluding F32.3 and F33.3	267	6
Substance-induced psychosis	F10-F19 with fourth digit 4, 5, or 7	267	6
Nonpsychotic alcohol use disorders	F10 excluding diagnoses with fourth digit 4, 5, or 7	237	5
Anxiety disorders	F40-F43	236	5
Bipolar disorder	F30-F31, F34.0	157	3

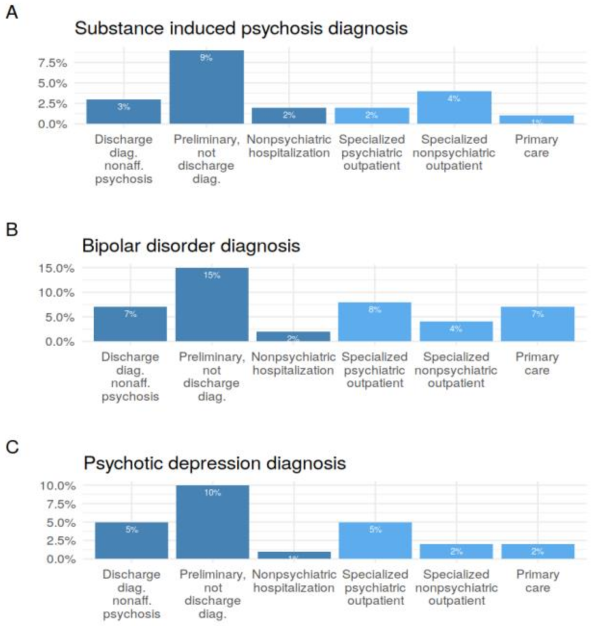

Suppelementary Figure 1. The probability of other psychotic diagnosis in people who had not received another nonaffective psychosis diagnosis by the treatment setting (hospital setting in dark blue and outpatient setting in light blue) of the first diagnosis

A

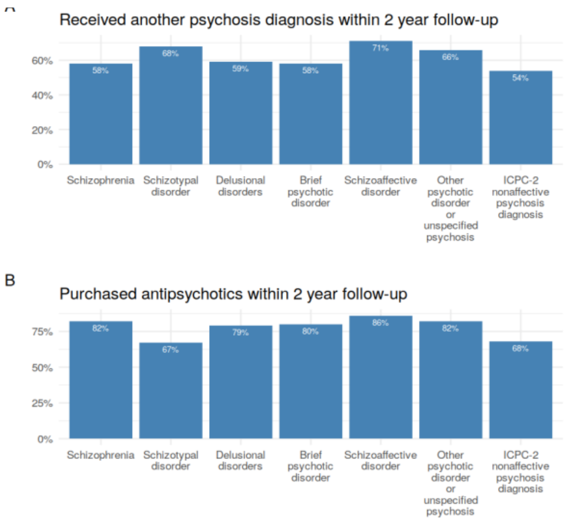

Suppelementary Figure 2. The probability of of receiving another psychosis diagnosis or purchasing antipsychotics with 2-year follow-up by the first nonaffective psychosis diagnosis.

Supplmentary Table 4. Logistic regression analyses predicting another nonaffective psychosis diagnosis and antipsychotic purchase with 2-year follow-up

A) Another nonaffective psychosis diagnosis at follow-up

	OR	95% CI	
Gender: woman	1,08	1,03	1,13
**Age**, reference 21-40			
7-20	0,98	0,92	1,06
41-60	0,67	0,63	0,71
61-70	0,60	0,56	0,65
71-80	0,50	0,47	0,55
81-90	0,35	0,31	0,38
91-115	0,21	0,17	0,26
**First diagnosis**, reference other or unspecified psychiatric disorder			
Schizophrenia	1,07	0,99	1,16
Schizoaffective disorder	1,21	1,06	1,37
Delusional disorders	1,18	1,10	1,26
Brief psychiatric disorder	0,82	0,77	0,88
Schizotypal disorder	0,99	0,87	1,13
ICPC-2 diagnosis of nonaffetive psychosis	1,02	0,93	1,12
**Treatment facility**, reference psychiatric outpatient care			
Discharge diagnosis from psychiatric hospitalization	0,81	0,76	0,86
Only preliminary diagnosis from psychiatric hospitalization	0,11	0,10	0,11
Nonpsychiatric hospitalization	0,20	0,18	0,21
Specialized nonpsychiatric outpatient care	0,28	0,25	0,32
Primary care	0,41	0,38	0,44

B) Antipsychotic purchase at follow-up

	OR	95% CI	
Gender: woman	1,21	1,15	1,27
**Age**, reference 21-40			
7-20	0,91	0,84	0,98
41-60	1,02	0,96	1,09
61-70	1,17	1,07	1,27
71-80	1,27	1,15	1,40
81-90	1,07	0,96	1,20
91-115	1,04	0,83	1,32
**First diagnosis**, reference other or unspecified psychiatric disorder			
Schizophrenia	1,24	1,13	1,37
Schizoaffective disorder	1,41	1,20	1,65
Delusional disorders	0,90	0,83	0,98
Brief psychiatric disorder	0,84	0,78	0,90
Schizotypal disorder	0,51	0,45	0,58
ICPC-2 diagnosis of nonaffetive psychosis	0,58	0,53	0,64
**Treatment facility**, reference psychiatric outpatient care			
Discharge diagnosis from psychiatric hospitalization	2,05	1,91	2,20
Only preliminary diagnosis from psychiatric hospitalization	0,82	0,76	0,89
Nonpsychiatric hospitalization	0,69	0,62	0,76
Specialized nonpsychiatric outpatient care	0,43	0,38	0,49
Primary care	0,75	0,70	0,82

Suppementary Table 5. The people with special reimbursement right before their first diagnosis in CRHC

	n	% of the whole cohort
n of people with special reimbursement right	9 810	20
Granted before 1996 (i.e. start of CRHC follow-up)	3878	8
Granted between 1996 – the first NAP diagnosis	5 937	12
The recorded diagnosis [1]	n	% of the people with the reimbursement right
NAP	3 639	37
Bipolar disorder	1 018	10
Psychotic depression	1 655	17
Other	214	2
No diagnosis recorded	3 284	33

[1] We included the first special reimbursement right for each individual, and the first recorded diagnosis entitling to the reimbursement. NAP diagnoses were defined as F20-F29 (ICD-10), 295, 297, 298 and 3012C (ICD-9), psychotic depression as F32.3 and F33.3 (ICD-10) and 2961E (ICD-9) and bipolar disorder as F30, F31 and F34.0 (ICD-10) and 2962, 2963, 2964 and 2967A (ICD-9). Other diagnoses were classified as 'other'.

Supplementary Methods: The description of the registers

Care Register for Health Care

The public health care in Finland is divided to special health care and primary health care. The Care Register for Health Care contains data special health care. The register was at first called Hospital Discharge Register, when it included only data on patients discharged from hospitals. The name was changed to Care Register for Health Care in year 1994 when the data content became more comprehensive. From year 1998, the register has included data on specialized psychiatric outpatient care. In addition, the data includes other specialized outpatient care, count of patients in inpatient care on 31 December each year as well as day surgeries. The register content of the register includes, for example basic data on service provider, patient, data on start and discharge from care, diagnoses, and interventions as well as additional data on psychiatric treatment, such as length of involuntary care.

The register controller is National Institute for Health and Welfare. More information of the register can be found at https://thl.fi/en/web/thlfi-en/statistics-and-data/data-and-services/register-descriptions/care-register-for-health-care.

Register of Primary Health Care visits

The register contains data from outpatient primary health care units from year 2011. The data content includes, for example basic information of the client, service provider, first contact with the provided, appointment data and data on the visit or on the cancellation of the appointment. The diagnoses assigned at primary health care can be based on International Classification of Diseases (ICD)-10 codes or International Classification of Primary Care (ICPC)-2 codes.

The register controller is National Institute for Health and Welfare. More information of the register can be found at https://thl.fi/en/web/thlfi-en/statistics-and-data/data-and-services/register-descriptions/register-of-primary-health-care-visits.

Register for Reimbursements for Prescription Medicines

The register includes prescribed outpatient medication use in Finland. All permanent residents of Finland are entitled to reimbursements for the cost of prescribed medicines. The register does not contain medications administered in hospitals. The data includes the basic information about the patient, the purchased medications, such as, Anatomical Therapeutic Chemical (ATC), number of packages and defined daily doses (DDD) and costs. The register was founded on year 1993, and the data is comprehensive from year 1995.

The register controller is Social Insurance Institution of Finland.

Register for Special Reimbursement Right

The register contains the special reimbursement rights granted to people from year 1964. Reimbursement right can be granted based on an illness that is considered to be serious and chronic. The right can be granted as continuous or for a fixed period. The diagnosis entitling to the

reimbursement was not systematically recorded in the early years of the register, and the information on diagnosis can be considered comprehensive from year 2000.

The special reimbursement right due to costs from medications for the treatment of severe mental disorder (# 112) can be granted to people with severe and long-term mental disorder, such as, schizophrenia, delusional disorder, bipolar disorder, psychotic depression, or dementia, Parkinson's, autism or related condition with psychotic features. Reactive psychoses do not entitle to the special reimbursement right. The right can be applied based on an examination by a psychiatrist.

The register controller is Social Insurance Institution of Finland. More information on medication reimbursement system in Finland can be found, for example at https://www.julkari.fi/bitstream/handle/10024/143552/Finnish_statistics_on_medicines_2020.pdf?sequence=1&isAllowed=y.

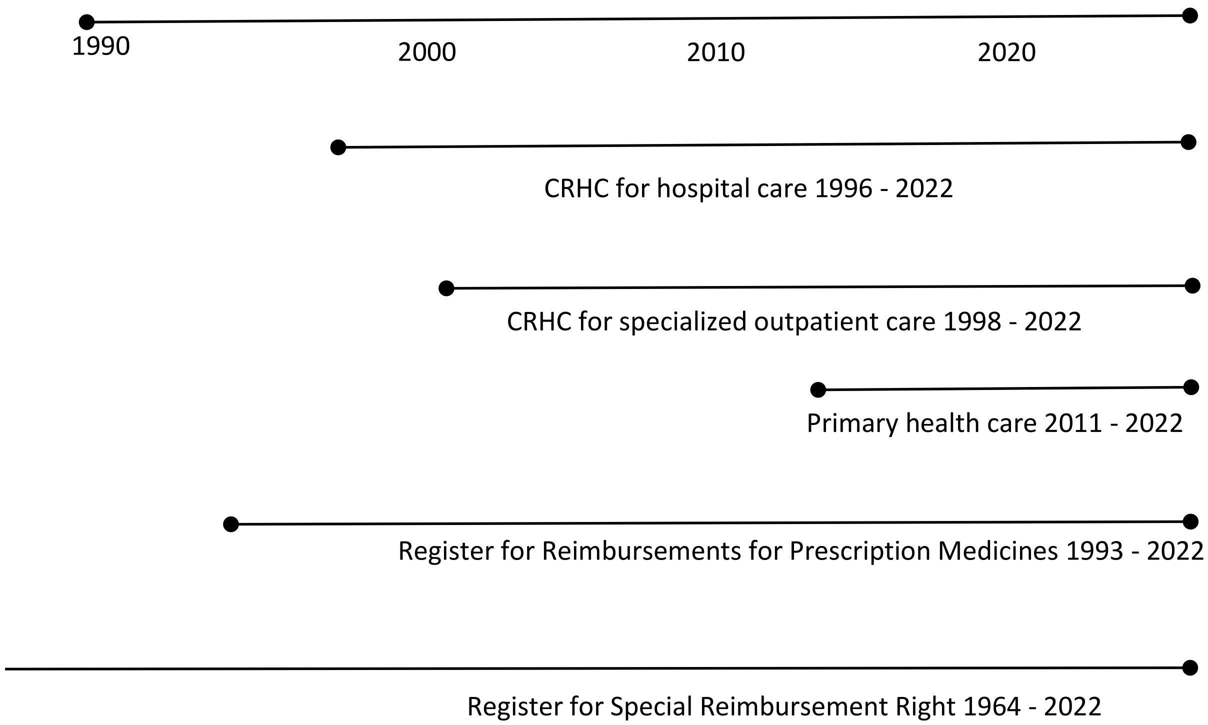

Figure 1. The timeframes when data from registers was available.
